# Multidisciplinary European Low Dose Initiative (MELODI): strategic research agenda for low dose radiation risk research

**DOI:** 10.1007/s00411-017-0726-1

**Published:** 2017-12-15

**Authors:** M. Kreuzer, A. Auvinen, E. Cardis, M. Durante, M. Harms-Ringdahl, J. R. Jourdain, B. G. Madas, A. Ottolenghi, S. Pazzaglia, K. M. Prise, R. Quintens, L. Sabatier, S. Bouffler

**Affiliations:** 1 Department of Radiation Protection and Health, Federal Office for Radiation Protection, BfS, Neuherberg, Germany; 2 University of Tampere, Tampere, Finland; 3 STUK, Helsinki, Finland; 4 ISGlobal, Barcelona Institute for Global Health, Barcelona, Spain; 5 Institute for Fundamental Physics and Applications, TIFPA, Trento, Italy; 6 Centre for Radiation Protection Research, Stockholm University, Stockholm, Sweden; 7 Institute for Radiological Protection and Nuclear Safety, IRSN, Fontenay-aux-roses, France; 8 Environmental Physics Department, MTA Centre for Energy Research, Budapest, Hungary; 9 Physics Department, University of Pavia, Pavia, Italy; 10 Italian National Agency for New Technologies, Energy and Sustainable Economic Development (ENEA), Rome, Italy; 11 Queens University Belfast, Belfast, UK; 12 Belgian Nuclear Research Centre, SCK-CEN, Mol, Belgium; 13 French Atomic Energy Commission, CEA, Paris, France; 14 Public Health England, PHE, Chilton, UK

**Keywords:** Low-dose, Health effects, Cancer, Non-cancer, Individual sensitivity, Ionizing radiation

## Abstract

MELODI (Multidisciplinary European Low Dose Initiative) is a European radiation protection research platform with focus on research on health risks after exposure to low-dose ionising radiation. It was founded in 2010 and currently includes 44 members from 18 countries. A major activity of MELODI is the continuous development of a long-term European Strategic Research Agenda (SRA) on low-dose risk for radiation protection. The SRA is intended to identify priorities for national and European radiation protection research programs as a basis for the preparation of competitive calls at the European level. Among those key priorities is the improvement of health risk estimates for exposures close to the dose limits for workers and to reference levels for the population in emergency situations. Another activity of MELODI is to ensure the availability of European key infrastructures for research activities, and the long-term maintenance of competences in radiation research via an integrated European approach for training and education. The MELODI SRA identifies three key research topics in low dose or low dose-rate radiation risk research: (1) dose and dose rate dependence of cancer risk, (2) radiation-induced non-cancer effects and (3) individual radiation sensitivity. The research required to improve the evidence base for each of the three key topics relates to three research lines: (1) research to improve understanding of the mechanisms contributing to radiogenic diseases, (2) epidemiological research to improve health risk evaluation of radiation exposure and (3) research to address the effects and risks associated with internal exposures, differing radiation qualities and inhomogeneous exposures. The full SRA and associated documents can be downloaded from the MELODI website (http://www.melodi-online.eu/sra.html).

## Introduction

Exposure to ionising radiation from natural and/or artificial sources is ubiquitous and unavoidable. Medical and natural sources represent the largest fractions of the average effective dose received by the general public (UNSCEAR 2008). Exposures to artificial sources can vary between individuals depending on their occupation (e.g. employment in the nuclear industry, in air transport and in medicine), their health status (leading to varying medical exposures) and in rare cases on their place of living (due to potential environmental contamination). In addition to the fact that ionising radiation is unavoidable and variable in the population, it is known to damage health at certain exposure levels. At very high doses (> 1 Gy whole body) ionising radiation can be acutely lethal, while it can induce tissue damage following more localized high-dose exposures (ICRP 2012, AGIR 2009). Fortunately, those exposures are very rare. However, tissue damage can occur in various organs following life-saving cancer radiotherapy, with prevalences of up to 15% of the patients depending on the treatment (Dörr and Hendry 2001; Paulino et al. 2010).

Since many decades it is known that radiation can cause cancer in humans following acute exposure in the dose range of a few Sv down to about 100 mSv. More recently, evidence has accumulated that these more moderate exposures may also contribute to other conditions such as circulatory disease, lens opacities and effects on future generations (hereditary effects) (UNSCEAR 2010). The cancer risks to humans are established down to about 100 mSv, while for circulatory diseases and lens opacities they are established down to about 500 mSv (AGIR 2010). Below these levels, especially following protracted or other non-homogenous exposures, the risks to human health are less certain. The current system of radiation protection developed by the International Commission on Radiological Protection (ICRP) aims to avoid tissue injury and minimize the probability for cancer and hereditary disease (ICRP 2007). Risks of cancer and hereditary effects below 100 mSv are regulated assuming a linear non-threshold (LNT) relationship between dose and effect; however, for those low-dose exposures there is a large uncertainty about the exact dose response, and the impact of protracting exposures over long periods such as employment from, around 18 years of age to retirement.

There are various sources of ionising radiation in modern society. Medical uses of radiation for diagnostics and therapy are becoming increasingly prevalent. Nuclear power generation is viewed by many as a low-carbon dioxide emitting efficient energy source, and industrial radiography plays important roles in safety assessment. High radon exposures in buildings are a major issue in many countries. Long-distance air travel can lead to increased exposures of aircrew and passengers. Other sources are exposures to naturally occurring radioactive materials (NORM) in the oil extraction and other industries.

Therefore, an appropriate and acceptable balance between the benefits of use of ionising radiation on the one hand and the potentially associated health risk on the other hand is indispensable. The protection of individuals and populations comes at a cost, and there are disadvantages of both under- and overprotection. This applies in all exposure situations—existing elevated exposure situations such as high radon concentrations in buildings, occupational exposure situations for example in nuclear industry and the medical sector, and accidental situations where difficult decisions on countermeasure implementation such as health monitoring, sheltering and evacuation are required. In all of these contexts, it is of utmost importance that robust and accurate information is available on the magnitude of health risks posed by any given radiation doses, ranging from high to low. The main uncertainties in radiation health risk evaluation are in the magnitude of cancer risk at low acute and protracted doses below 100 mSv, the magnitude of non-cancer effects below 500 mSv and the variation in disease risk between individuals in the population. These are the key areas identified by Multidisciplinary European Low Dose Initiative (MELODI) requiring further research to provide better and more solid evidence for appropriate decision making in all areas of radiation protection. Accurate and reliable health risk estimation of low dose and dose-rate exposures of humans is an essential foundation for a robust and acceptable system of radiation protection.

### The European research platform MELODI

The purpose of the MELODI association is to establish a European research platform with a focus on health risk assessment after exposures to low-dose ionising radiation and its application for radiation protection, aiming for a progressive integration of national and European activities (http://www.melodi-online.eu/) (Repussard 2015). MELODI was founded in 2010 as a registered association with 15 institutional members. As of July 2017, MELODI has 44 members from national bodies responsible for defining, funding and implementing radiation research, and universities and research institutes committed to contributing to research and development in this domain. MELODI contributes to the setting of priority objectives in low-dose risk research, to the establishment of research programs and identification of resources to be implemented in order to achieve these objectives. Furthermore, MELODI supports the assessment of results obtained, education and promotion of communication on these issues between the various parties involved as well as sustainability of key research activities (Salomaa et al. 2015, 2017; Belli et al. 2011, 2015). Major actions of MELODI include organization of scientific and stakeholder workshops, promotion of the visibility of the research area, nomination of working groups on specific topics and facilitating collaborative research.

The establishment and regular updating of a long-term (> 20 years) Strategic Research Agenda (SRA) for research on low-dose health risk radiation protection in Europe is a key activity of MELODI. This SRA is supposed to provide guidance on the priorities for national and European research programmes and on the preparation of competitive calls at the European level. Furthermore, MELODI supports the availability of key infrastructures in Europe as an essential basis for research activities, and the long-term maintenance of competences in radiation research and health risk assessment by means of an integrated European approach for training and education. Consequently, MELODI established three working groups (WGs): one on the MELODI SRA, one on education and training, and a third on infrastructures.

The European Network of Excellence DoReMi (2010–2016) funded by Euratom FP7 Radiation Protection Programme served as an important initial operational tool for establishing MELODI (Salomaa et al. 2015). Currently, major parts of European radiation protection research are being organized within the CONCERT European Joint Programme Co-fund Action (EJP). CONCERT brings together funding agencies from the European Commission (EC) and the member states in an effort to integrate European radiation protection research and launches research calls in radiation protection on behalf of the EC. An important operational tool for the establishment of CONCERT was the European project OPERRA (Open Project for European Radiation Research Area, 2013–2017). Integration will build upon the SRAs from five European radiation protection research platforms, MELODI, ALLIANCE (radioecology) (Hinton et al. 2013), NERIS (emergency management) (Schneider et al. 2016), EURADOS (dosimetry of ionising radiation) (Rühm et al. 2016) and EURAMED (medical exposures) (EURAMED 2017), and aims to establish interaction and synergies between these different areas of expertise (Salomaa et al. 2017).

### MELODI SRA

The MELODI SRA has been updated yearly since 2010, taking into account results of ongoing, completed and published research, and new key radiation protection research issues, which may have arisen since the previous update. The procedure of SRA development includes that an updated draft and a short MELODI statement (only in years where a call will be launched), presenting the top priorities, is posted on the public MELODI website 6–8 weeks before the annual MELODI workshop takes place. Those interested (scientists, stakeholders) can then join an open consultation process on the MELODI website and/or participate in the following MELODI workshop to provide input before the SRA’s and statement’s revision. The first draft of a MELODI SRA was published in October 2010. Currently the eighth draft of the MELODI SRA is available on the MELODI website. All published MELODI SRAs can be downloaded from http://www.melodi-online.eu/sra.html. Summaries of the MELODI workshops are published (Salomaa et al. 2013; Aerts et al. 2014; Bouffler 2017) or can be found through the MELODI website.

The MELODI SRA has been developed following the key policy goals, to address the robustness of the current radiation protection system, as defined by the High Level Expert Group on European Low Dose Risk Research (HLEG) (http://www.hleg.de/). These goals include research on: (a) the shape of the dose–response for cancer, (b) tissue sensitivities for cancer induction, (c) individual variability in cancer risk, (d) the effects of radiation quality type, (e) risks from internal radiation exposure, and, (f) risks of, and dose response relationships for, non-cancer diseases and hereditary effects.

For the purpose of the MELODI SRA, these issues were restructured into three key topics low-dose and dose-rate radiation health risk research:

1. Dose and dose-rate relationship for cancer;
2. non-cancer effects; and
3. individual radiation sensitivity.

As discussed by the HLEG and confirmed by the DoReMi Network of Excellence and MELODI, research at low dose-rates or low doses includes major challenges in the investigation of both radiation-related health effects and underlying biological mechanisms, because the magnitude of health risk and biological effects is expected to be low. A multidisciplinary approach is therefore essential. For this reason, each key topic is sub-divided into three research lines:

- Research to improve understanding of the mechanisms contributing to radiation risk following low dose/dose-rate exposures.
- Epidemiological research that integrates, where possible and informative, biological approaches to improve health risk evaluation of radiation exposure.
- Research specifically aimed to address the effects of and risks associated with internal exposures, different radiation qualities and inhomogeneous exposures.

In the following text, the three key research topics are described and the research needed for an improvement of the evidence base for each of these topics is described based on the above-mentioned three research lines. In Tables 1, 2 and 3 the priority research areas within each key topic and research line are given.

**Table 1:** Priority research areas for the key research topic: “dose and dose rate dependence of cancer risk at low doses or dose rates” within three research lines

Basic mechanisms	Health risk evaluation	Impact of radiation exposure characteristics
•To determine the nature of the target cells for radiation carcinogenesis •To determine the contribution of DNA damage/mutational processes •To determine the contribution of epigenetic modifications •To determine the influence of cell micro-environmental, non-targeted and systemic processes •To examine the extent to which any of the above are different at high dose/dose-rate by comparison with low dose/dose-rate	•To determine the shape of the dose and dose-rate response relationship for total cancer based on key informative epidemiological studies •To determine the risk for different cancer sites based on key cohorts (⟹ tissue sensitivity) •To identify and validate biomarkers of exposure and health effects related to cancer •To evaluate cancer risks through systems biological analyses and models of carcinogenesis based on mechanistic studies and epidemiological data, and integration of the two •To collect tumour tissue for the molecular characterization of tumours and the study of dose–response in relation to tumour type •To investigate pre-stages of cancer in tissue or blood in order to allow modelling carcinogenesis •To identify human population studies where hereditary effects could be observed, if present	•To determine the cancer risk related to internal emitters in epidemiological studies, incorporating detailed dosimetric assessment and evaluation of dosimetric uncertainties, and—where appropriate—microdosimetric considerations •To conduct experimental studies in *vivo* or in *vitro* to test exposure scenarios where dose modulation plays a role •To describe by complex systems biology and bio-mathematical approaches the role of spatial inhomogeneity of radiation exposure in cellular, tissue, and organ levels in case of internal exposure of high LET radiation •To determine the Relative Biological Effectiveness (RBE) for selected endpoints in innovative experimental and epidemiological studies for specific cancer sites through comparison of risk related to low- and high-LET radiation

**Table 2:** Priority research areas related to the key research topic: “non-cancer effects at low doses or dose rates” within three research lines

Basic mechanisms	Health risk evaluation	Impact of radiation exposure characteristics
•To develop in vitro and animal models of radiation-related non-cancer diseases in order to clarify the regulatory pathways involved •To apply a full range of analytical methods including ‘omics technologies’ and consideration of the target cells and surrounding microenvironment •To determine the contribution of radiation-related changes in the immune function and inflammatory processes in the pathogenesis of non-cancer effects	•To determine the shape of the dose-rate and dose–response relationship in humans for non-cancer outcomes at low or moderate doses based on key informative epidemiological studies •To identify, develop, and validate biomarkers for exposure, early and late effects •To evaluate non-cancer risk through systems biological analyses and mathematical models combining mechanistic studies and epidemiological data, and integration of the two •To investigate early stages in the progression of non-cancer effects in tissue or disease-related endpoints in biological samples in order to understand spontaneous pathogenesis	•To investigate the biological mechanisms that govern the effects observed in tissues involved in non-cancer effects after low-dose exposure regarding specific exposure modalities and radiation qualities •To identify clinically relevant pathways involved in low-dose radiation-induced non-cancer effects via systems biology (linked to nano- and/or microdosimetry) •To conduct epidemiological studies of internal emitter risk •To develop new and innovative ways in experimental studies to determine the Relative Biological Effectiveness (RBE) using up-to-date technologies and to determine/compare the effects of acute versus chronic exposure

**Table 3:** Priority research areas related to the key research topic: “individual radiation-sensitivity” within three research lines

Basic mechanisms	Health risk evaluation	Impact of radiation exposure characteristics
•To develop an understanding of the pathways affected by acute and long-term responses to low doses of radiation (inflammatory processes and immunological states) •To use systems biology and modelling to predict differences in outcome at both individual (qualitative changes affecting health-relevant pathways) and population (quantitative changes in health outcomes) levels •To identify genetic and epigenetic biomarkers of susceptibility to radiation-associated disease that can be applied in molecular epidemiology •To investigate mechanisms by which age at exposure, attained age, sex and lifestyle and other factors, including co-exposures to other agents may affect radiation risk	•To validate candidate biomarkers of individual sensitivity identified from the mechanistic studies in cohorts of exposed and non-exposed subjects that have developed cancer or non-cancer diseases •To improve or set-up key cohorts and conduct molecular epidemiological studies to determine factors (host and environmental) involved in individual sensitivity to radiation-induced cancer and non-cancer effects and to quantify their effects •To quantify the variation in risk between different population groups and the impact of different factors (age at exposure, attained age, co-exposures, and host factors) •To develop systems biology models of radiation-induced pathogenesis in dependence on individual risk factors	•To develop suitable cell, tissue, and in vivo models for the quantification of the impact of dose inhomogeneity and radiation quality on individual radio-sensitivity •To conduct epidemiological studies for the quantification of the impact of dose inhomogeneity and radiation quality on individual radio-sensitivity •To characterize how internal exposure, dose inhomogeneity, and radiation quality will influence the formation of candidate biomarkers identified in response to low LET external exposure •To study how dose distributions and related biological effects can vary between individuals at the same exposure conditions because of anatomical and physiological differences

## Key research topic 1: “dose and dose-rate-dependence of cancer risk”

Current risk estimates used in radiation protection are based upon epidemiological studies of exposed populations (UNSCEAR 2008; Boice 2017). Radiation protection standards aim to avoid tissue reactions and minimize the incidence of the late developing stochastic effects of cancers and hereditary effects in future generations (ICRP 2007). Thus, for radiological protection it is of fundamental importance that the health risk estimates are robust and credible. Most important among the epidemiological studies are the follow-up studies on Japanese A-bomb survivors of Hiroshima and Nagasaki that provided clear evidence of increased cancer risk (Ozasa et al. 2012; Grant et al. 2017). While these studies remain the main basis for the cancer risk estimates used in radiation protection, they relate to a specific population and a specific exposure scenario. The exposure was essentially an acute, high dose-rate total-body gamma ray exposure with a small neutron component. Therefore, the A-bomb survivor studies are to an increasing extent complemented by occupational, environmental, and medical exposure studies (UNSCEAR 2008; Shore et al. 2017), which allow direct investigation of effects of fractionated or more protracted exposures and effects of lower doses (Rühm et al. 2015). Evidence for radiation-related hereditary effects is based on experimental animal studies because there is no direct evidence from human studies to date. The contribution of hereditary risk to the overall risk is estimated to be small in comparison with cancer risk (ICRP 2007).

Epidemiological studies provide evidence of dose-related increases in total cancer risk after acute exposures with doses of about 100 mSv and above. Recent pooled occupational studies suggest increased solid cancer and leukaemia risks following protracted radiation exposures of the order of around 100 mSv (Richardson et al. 2015; Leuraud et al. 2015). Further, recent reports indicate a possible association between natural background gamma radiation exposures and risk of childhood leukaemia (Spycher et al. 2015) and suggest an elevated risk associated with medical imaging methods (Walsh et al. 2014).

Nevertheless, there are major uncertainties concerning (1) the magnitude of cancer risk following protracted exposures encountered in the environment, in medicine and in occupational settings, particularly those of the order of 100 mSv or less; (2) organ-specific risks following acute or protracted doses of a few 100 mSv, particularly for inhomogeneous dose distributions; (3) the risk for specific cancer sites due to possibly different tissue sensitivities, and (4) the most scientifically evidence-based model to infer risks at doses and dose-rates lower than those for which direct epidemiological evidence is available. In this context, there are also a number of ethical questions that need to be addressed, such as “precautionary” use of the LNT model for extrapolation to doses far below those where risk estimates are considered well founded on empirical observations.

Classical epidemiological studies will need to be continued to refine the direct knowledge of risk in human populations, particularly in the context of low-dose and protracted exposures, and internal contamination. Biologically-based mechanistic and epidemiological approaches should be combined to quantify cancer risks from acute whole body exposures with low dose (< 100 mSv) or from protracted or inhomogeneous exposures at low to moderate doses (a few 100 mSv or less). They also need to address the impact of different radiation qualities and effects of both internal and external exposures, alone and in combination. Knowledge of health risks from such low dose-rate exposures is of key relevance for planning measures in emergency situations, particularly for large populations and for radiation protection of occupationally exposed persons, to evaluate the present dose limit of 20 mSv/year averaged over 5 years with no single year exceeding 50 mSv (EURATOM 2014).

### Research line 1: basic mechanisms

An LNT extrapolation model is currently used in radiation protection to estimate risk at low doses from epidemiological data obtained at higher doses (ICRP 2007; Boice 2017). An important aspect for the justification of using this model is that radiation carcinogenesis is assumed to be primarily driven by damage to DNA and subsequent mutations of growth-regulating genes in target cells (Brenner et al. 2003). Yet, a number of other potential mechanisms contributing to and modulating radiation carcinogenesis have been proposed (UNSCEAR 2012) and it is important to determine their potential roles (Kadhim et al. 2013). The extent to which these modulations and non-mutational mechanisms support or challenge the validity of a LNT risk extrapolation model needs to be determined under relevant exposure conditions. For this purpose, the use of well-validated animal and human cellular/tissue models of radiation carcinogenesis (both solid cancers and leukaemias) is required.

### Research line 2: health risk evaluation

Quantification of cancer risk at moderate dose or dose-rates from inhomogeneous or protracted exposure, and at low doses or dose-rates from acute, homogenous exposure is a key challenge. The large size of epidemiological studies required to detect small increases in cancer risk at low doses and dose-rates and the potential for bias and confounding present challenges, particularly at the lowest doses (Brenner et al. 2003). The priorities in this area include the maintenance and improvement of key cohorts by continued follow-up, pooling of different studies, collection of information on confounders and reducing misclassification of dose and health data. Key cohorts are characterized by large populations with exposure conditions and dose distributions that are relevant for radiation protection, good individual dosimetry, long and complete follow-up with good quality data of health outcome, particularly on cancer occurrence; and the possibility of collecting information on relevant potential confounders either on the whole cohort or through nested case–control studies.

These studies should include, where possible and likely to be informative, the collection and appropriate storage of a large number of relevant biological samples, including tissue samples from most of the cancer cases. Through identification and integration of relevant biological endpoints and markers into epidemiological studies (Pernot et al. 2012; Hall et al. 2017), further insights will be gained into the risks associated with such exposures. The integration of both epidemiological and mechanistic studies will improve cancer risk evaluation through molecular epidemiological studies or by mechanistic modelling.

### Research line 3: impact of radiation exposure characteristics

It is important but often overlooked that radiation exposure in the environment, in occupational and medical settings can involve internal contamination, often to radiations of differing quality, or involve other aspects of dose inhomogeneity. The current system of protection uses radiation weighting factors to reflect spatial dose distribution differences between radiations of differing quality. The actual risks associated with all forms of dose inhomogeneity including those due to inhomogeneous activity distribution within a tissue like in case of radon exposure (Madas 2016) are not well understood. The extent to which these factors modify dose–response relationships for cancers is therefore important to understand.

## Key research topic 2: “non-cancer effects”

It has been traditionally assumed that health effects other than cancer and hereditary diseases show a threshold at doses that are above the levels of exposures typically encountered in the public environment, at work or from diagnostic medical uses of ionising radiation (ICRP 2012). Recent results from epidemiological and experimental studies indicate increased risks from vascular diseases (Little 2016; AGIR 2010), lens opacities (Shore 2016), cognitive/neurological effects (Verreet et al. 2016; Hall et al. 2004); and others not only at doses above 5 Gy but also at a range of doses from 5 down to 0.5 Gy and, possibly even at lower doses (< 0.5 Gy) (Shore 2014). Based on these findings the International Commission on Radiological Protection (ICRP) issued in 2011 a statement on tissue reactions (formerly termed non-stochastic or deterministic effects) that noted evidence that the threshold in absorbed dose for effects on the lens of the eyes is in the order of 0.5 Gy (acute and protracted exposure). Consequently, a recommendation was made for a reduction in the annual equivalent dose limit for the lens of the eye to 20 mSv per year averaged over 5 years with no 1 year exceeding 50 mSv (ICRP 2012). In addition, ICRP suggested that the absorbed dose threshold for circulatory diseases may be as low as 0.5 Gy. ICRP defines the threshold as the dose that causes the disease in 1% of the exposed persons.

For all outcomes, uncertainties and concerns exist about possible effects at low doses, which could have important implications for radiation protection. Results of epidemiological studies are not always consistent, as bias and confounding cannot be excluded, and the biological mechanisms of relevance for health risks at these low doses are not known. The possibility of a stochastic nature of non-cancer effects without dose thresholds raises a wide range of questions, and needs further investigation. In contrast to cancer and hereditary effects, knowledge on the underlying biological mechanisms for radiation-related non-cancer effects in the moderate and low-dose range is very sparse. Therefore, research to understand the mechanisms is necessary. In addition, careful epidemiological research based on key cohorts, integrating—where possible and informative—biological approaches is needed to provide information on radiation-related risk of non-cancer diseases following low-dose, protracted or fractionated exposure, relevant for radiation protection. Individual radiation sensitivity, mixed exposures and the impact of characteristics of radiation exposure also need to be considered.

### Research line 1: basic mechanisms

Deterministic effects or tissue reactions are classically thought to arise as a consequence of cell killing or functional inactivation by the (generally) high radiation doses involved (ICRP 2012). They are characterised by steeply increasing dose–response relationships at doses beyond a defined threshold. It is unlikely that cell killing/inactivation will be the only basis for effects of lower radiation doses in relation to vascular disease, cataract and cognitive dysfunction. Epidemiological investigations of populations with well-characterised exposures require studies to identify the underlying mechanisms that lead to each of the non-cancer disease. Each disease may have a different mechanistic basis, and it is not clear if there will be any similarity with the mechanisms that lead to radiation-related cancers.

### Research line 2: health risk evaluation

Quantification of non-cancer risk (circulatory diseases, lens opacities, others) in humans at moderate or low doses or dose-rates is a key, yet difficult challenge for radiation protection, because the magnitude of risk due to radiation is expected to be low and the potential for bias and confounding is high. Informative epidemiological studies in this field should be characterized by large cohorts with exposure scenarios and dose values of interest for radiation protection, good dosimetry, high quality of health data, long-term follow-up and the possibility of collecting information on relevant potential confounders either on the whole cohort or through targeted nested case–control studies. In addition, these studies should include—where possible and informative—collection of a large number of biological samples, relevant tissue samples from most cases in a given organ, and extensive data on the health status during follow-up.

Through improvement of key epidemiological studies (e.g., increasing the statistical power by pooling studies using standardized study protocols; improvement of appropriate organ and tissue dose assessment, e.g. different parts of the heart, main arteries and veins as well as blood, brain, eyes, lens,…) further insights will be gained into the risks associated with such exposures. In addition, where possible and informative, relevant biological endpoints and markers should be identified and integrated into epidemiological investigations (Kreuzer et al. 2015).

### Research line 3: impact of radiation exposure characteristics

Dose fractionation and dose-rate effects have been observed for the induction of non-cancer effects, e.g. low dose-rate dependent effects (premature senescence) seen in endothelial cells of the cardiovascular system (Yentrapalli et al. 2013; Rombouts et al. 2014). The impacts of radiation quality and inhomogeneous exposure for non-cancer endpoints are not well understood. If non-cancer diseases are included in the estimation of health risk at low doses, the radiation weighting factors applied may not be the same as those used for cancers and hereditary effects. The underlying mechanisms and their response to differing conditions of irradiation will need to be understood.

## Key research question 3: “individual radiation sensitivity”

Variability in radiation-related health risk and genetic susceptibility to radiation effects within a population is an important issue for radiation protection. Differences in radiation sensitivity between individuals, or groups, may relate to gender, age at exposure, attained age, state of health, genetic and epigenetic make-up, lifestyle, and co-exposures. Such differences, if significant, raise the ethical and policy question as to whether some individuals or groups are inadequately protected by the present system and regulations, and whether it would be acceptable to apply different exposure limits for various subgroups of the population or ultimately at the individual level.

At present, there is insufficient information to establish how large the differences in sensitivity may be between individuals or between groups of individuals and their consequent influence on risk estimates at low doses and dose-rates. In order to address policy questions it is necessary to obtain better knowledge on the extent of the variations in sensitivity in the population, both in the sizes of the variations and in the proportions of the population that are affected. This needs to include the impact of dose inhomogeneity, radiation quality, and internal versus external exposures. In addition, the nature of the interaction of ionising radiation with co-exposures to other agents (e.g. tobacco smoke, heavy metals) for various cancers is important in considering risk transfer between different populations.

### Research line 1: basic mechanisms

Basic research is needed to establish which factors and processes predispose individuals to greater risk of late effects in terms of cancer or non-cancer diseases. This includes both molecular epidemiological approaches, the discovery of genetic, phenotypic and molecular markers of these pathways (Hall et al. 2017; Pernot et al. 2012), and the integration of mechanistic studies in the quantitative evaluation of health risks. A major focus should be the understanding of how these different factors may modify risk keeping in mind that the radiosensitive phenotype is likely to be multifactorial. Another important question is whether acute or late markers of radiation sensitivity (adverse healthy tissue or organ responses after radiotherapy) are related to risk of developing late effects following exposure to low and protracted doses of different linear energy transfers (LETs) including internal exposures.

### Research line 2: health risk evaluation

The quantification of the contribution that individual sensitivity makes to radiation risk on both an individual and population level is a key question. Realistic estimates of how large the differences may be in extreme cases as well as the spread of sensitivities in average population groups will need large informative epidemiological studies with reliable information on potential effect modifiers and inclusion of molecular biomarkers. Systems biological analyses and mechanistically based models of disease are further prerequisites.

### Research line 3: impact of radiation exposure characteristics

The impact of external versus internal emitters, dose inhomogeneities and radiation quality on individual radio-sensitivity related to different doses and dose-rates has not been defined for relevant environmental, medical and occupational exposures. In case of internal contamination, individual radio-sensitivity could be dependent on localized dose distributions, but currently no mechanistic understanding, relevant experimental models, or valid datasets exist for these relationships. Similarly, radiation quality is gaining importance because of the more wide-spread availability of external beam hadrontherapy, where scattered neutrons are of concern, and the increasing clinical use of radionuclides (Belli et al. 2010).

Individual sensitivity should be analysed as a function of exposure and not only dose, because the same exposure can result in very different doses and spatial dose distributions in different individuals. For internal exposure, the dose distributions can be very different between individuals due to anatomical and physiological differences (e.g. airway morphology variability, different thickness of mucus layer in the bronchi or nose as opposed to mouth breathing). This variability should be taken into account and modelled for future analysis. Both accurate dosimetric models and physiologically relevant biokinetic models are required for the interpretation of the health and biological effects of internal emitters, especially for the characterization of individual sensitivities. There is also a need to better characterize how candidate biomarkers, identified in response to low LET (linear energy transfer) external exposure, may be influenced by internal exposure, dose inhomogeneity, radiation quality. In many situations, mixed field exposures are relevant but again there are no data related to the role of individual radio-sensitivity.

## Education and training (E&T)

The HLEG report of 2009 (http://www.hleg.de/fr.pdf) identified a problem with the maintenance in Europe of the range of expertise essential to an effective programme of research into the risks to humans from low-dose radiation. The report advised that specific programmes aiming at knowledge management across generations need to be designed in order to achieve sustainable continuity and development.

It is important that sponsored courses and workshops are provided to attract students into the radiation research area. A successful example is the annual series of short courses funded by CONCERT (http://www.concert-h2020.eu/en/Concert_info/Education_Training). A large proportion of the groundwork of research is carried out as student projects and thesis work. For this reason, the research effort relies on a continuing relationship with universities, and on a healthy stream of high-level students. It is, therefore, essential that this symbiosis is recognised and taken into account in research funding structures.

A further role of E&T within any specialized research area is in dissemination of new technologies, skills, and knowledge. To obtain maximum impact and benefit from research there should be a programme of workshops, seminars, summer schools, etc. which is integrated into the design and funding structure of all research. The programme should be aimed both at sharing knowledge within the European low-dose research community and at the wider radiation protection field including radioecology, emergency response, and the medical use of radiation.

Because the number of centres of expertise in low-dose research in Europe is limited, it is important that the centres collaborate to provide a coordinated E&T programme. University courses should be modular, and mutual recognition agreements should permit students to transfer across EU states to construct degree courses. There is a need for a forum to facilitate networking and to bring together all interested parties regularly to discuss needs and broaden the awareness of what is happening in EU member states.

## Infrastructures

One of the roles of MELODI is to ensure the availability of and to facilitate ready access to the state-of-the-art research infrastructures required to support the research efforts in low dose research. The priority is to promote the use of mature and up-to-date infrastructures and avoid unnecessary duplication. Furthermore, an effort should be made to harmonize practices amongst multiple facilities. Finally, the sustainability of rare but necessary facilities (e.g. for internal contamination) needs to be guaranteed. This should include recommendations on the provision of the financial means to harmonize, sustain and access these facilities.

Infrastructures include so-called large infrastructures such as exposure facilities including those for animal experimentation, as well as the collection and storage of cohort data, databases, biobanks, and analytical platforms.

Within the EU-funded project DoReMi, an extensive list of relevant infrastructures was generated for low-dose research in particular irradiation facilities for internal and external exposure. Concerning relevant epidemiological cohorts, priority should be given to cohorts and biobanks that permit studies to improve the quantification of the risk associated with low dose and low dose-rate radiation exposure, for cancer and/or non-cancer diseases and/or to identify groups of individuals with specific sensitivity. In the short-term, existing epidemiological cohorts can be used to support modeling and/or molecular studies for which the requirements differ. In the long-term, new prospective cohorts can also be envisaged, as well as the development of new collections of biological material. Analytical platforms that can screen a large set of validated biomarkers (such as the European dosimetry network RENEB) are accessible for the rapid and reliable assessment of radiation exposure (Kulka et al. 2017).

Within the EU-funded project STORE (https://www.storedb.org/store_v3/), an internet-based platform for sharing data from epidemiological studies, as well as data and biological samples from radiation experiments (new and past), has been developed, further carried forward and supported first by DoReMi then by CONCERT. Going forward, it will be necessary to promote activities to further update and continuously expand the content of the database, and to elucidate to what extent data from other radiation protection platforms (ALLIANCE, NERIS and EURADOS) can be incorporated into STORE or whether a comparable database would be more appropriate.

Activities to identify valuable materials and archives that could be included in the database and the tissue bank, as well as to maintain relevant biobanks and rescue material from endangered biobanks, need to be supported. The maturation of the so-called ‘omics technologies’ and systems biology may offer novel opportunities for European radiation protection research. In order to benefit from the best quality of technologies and supporting managerial and technical support, efforts should be given to facilitating access to major national and European infrastructure platforms.

Priority areas related to infrastructures are: (1) improvement of the access to infrastructures, (2) favor open access to radiation research data within STORE, (3) re-use of archived materials using specific retrospective approaches, (4) enlargement and sustainability of RENEB, including inter-comparison exercises, and (5) improvement of the knowledge of existing infrastructure via E&T courses.
